# Establishing plausibility of cardiovascular adverse effects of immunotherapies using Mendelian randomisation

**DOI:** 10.3389/fcvm.2023.1116799

**Published:** 2023-05-19

**Authors:** Nhu Ngoc Le, Tran Quoc Bao Tran, Clea du Toit, Dipender Gill, Sandosh Padmanabhan

**Affiliations:** ^1^ School of Cardiovascular and Metabolic Health, University of Glasgow, Glasgow, United Kingdom; ^2^ Department of Epidemiology and Biostatistics, School of Public Health, Imperial College London, London, United Kingdom

**Keywords:** GWAS, pleiotropy, Mendelian randomisation, reactome, eQTL, stroke, diabetes, blood pressure

## Abstract

Immune checkpoint inhibitors (ICIs) and Janus kinase inhibitors (JAKis) have raised concerns over serious unexpected cardiovascular adverse events. The widespread pleiotropy in genome-wide association studies offers an opportunity to identify cardiovascular risks from in-development drugs to help inform appropriate trial design and pharmacovigilance strategies. This study uses the Mendelian randomization (MR) approach to study the causal effects of 9 cardiovascular risk factors on ischemic stroke risk both independently and by mediation, followed by an interrogation of the implicated expression quantitative trait loci (eQTLs) to determine if the enriched pathways can explain the adverse stroke events observed with ICI or JAKi treatment. Genetic predisposition to higher systolic blood pressure (SBP), diastolic blood pressure (DBP), body mass index (BMI), waist-to-hip ratio (WHR), low-density lipoprotein cholesterol (LDL), triglycerides (TG), type 2 diabetes (T2DM), and smoking index were associated with higher ischemic stroke risk. The associations of genetically predicted BMI, WHR, and TG on the outcome were attenuated after adjusting for genetically predicted T2DM [BMI: 53.15% mediated, 95% CI 17.21%–89.10%; WHR: 42.92% (4.17%–81.67%); TG: 72.05% (10.63%–133.46%)]. JAKis, programmed cell death protein 1 and programmed death ligand 1 inhibitors were implicated in the pathways enriched by the genes related to the instruments for each of SBP, DBP, WHR, T2DM, and LDL. Overall, MR mediation analyses support the role of T2DM in mediating the effects of BMI, WHR, and TG on ischemic stroke risk and follow-up pathway enrichment analysis highlights the utility of this approach in the early identification of potential harm from drugs.

## 1. Introduction

Whilst clinical trials are the gold-standard for establishing efficacy and short-term safety of drugs, they are not ideal for identifying long term treatment-related adverse effects and have limited generalisability.

Newer therapies such as immune checkpoint inhibitors (ICIs) and Janus kinase inhibitors (JAKis), which have transformed cancer immunotherapy and treatment of chronic inflammatory disorders, have recently raised concerns because of unexpected life-threatening cardiovascular adverse events (CVAEs) post-approval (1–5).

ICIs are monoclonal antibodies targeting cytotoxic T lymphocyte antigen-4 (CTLA-4), programmed death protein 1 (PD-1) and its ligand PD-L1, and lymphocyte-activation gene 3 (LAG-3). The result is a blockage of immune regulatory interactions, increased T cell activation, and antitumor immune response. Currently approved ICIs in clinical use have been shown to be negative regulators of atherosclerosis in animal and in-vitro studies through T cell inhibition, regulatory T cell differentiation and T cell exhaustion. Endothelial PD-L1 reduces apoptosis and can drive overexpression of tight junction molecules both of which stabilise atherosclerotic plaques (2). Indeed, ICIs are associated with a threefold higher risk for atherosclerotic cardiovascular events, including myocardial infarction, coronary revascularization, and ischemic stroke (1, 2). ICI treatment has been associated with increased cardiovascular adverse events with rates of stroke, heart failure, atrial fibrillation and conduction disorders ranging between 1.5% and 5% (6).

JAKis are used in the treatment of chronic inflammatory disorders and are immune modulating drugs that inhibit the Janus kinase/signal transducer and activator of transcription (JAK/STAT) signalling pathway. Clinical trials of the JAKi tofacitinib showed increased lipid levels and cancer incidence in treated patients and a subsequent trial found an increased risk of major adverse cardiovascular events (MACE) with tofacitinib compared to a Tumor necrosis factor (TNF) inhibitor (7). Consequently, tofacitinib, baricitinib, and upadacitinib now carry black box warnings about the risks of serious heart-related events, cancer, blood clots, and death (4, 5). Any method that can flag potential adverse events, such as those now associated with ICIs and JAKis, can help inform the trial design to ensure that these events are captured with adequate sample size and follow-up.

Pleiotropy is a widespread feature in genome-wide association studies (GWAS) and may help explain the unexpected adverse events related to newer therapies without elucidating the specific molecular mechanisms at play. In this study, we used Mendelian randomisation (MR) to study the causal effect of cardiovascular risk factors on the risk of ischaemic stroke both independently and by mediation, followed by interrogation of the implicated expression quantitative trait loci (eQTLs) to determine if the enriched pathways can explain the adverse stroke events observed with ICI or JAKi treatment.

## 2. Methods

The data used in this study are publicly accessible and were obtained with appropriate patient consent and ethical approval. The source studies are cited.

### 2.1. Two-sample MR

The MR analysis was reported in accordance with the STROBE-MR guidelines (Supplementary checklist in the Data Supplement) (8).

#### 2.1.1. Genetic association estimates

GWAS summary statistics for systolic blood pressure (SBP) and diastolic blood pressure (DBP) were derived from a GWAS meta-analysis for BP traits on 757,601 individuals of European ancestry from UK Biobank and the International Consortium of Blood Pressure Genome-Wide Association Studies (ICBP) (9). Genetic data for body mass index (BMI) and waist-to-hip ratio (WHR) were obtained from the GIANT Consortium GWAS meta-analysis of 806,834 and 697,734 European-ancestry individuals, respectively (10). GWAS summary statistics for low-density lipoprotein cholesterol (LDL) and triglycerides (TG) were derived from the Global Lipids Genetics Consortium GWAS of 1,320,016 individuals of European ancestry (11). Genetic association estimates for lifetime smoking (which captured smoking initiation, duration, heaviness, and cessation) were derived from a GWAS of 462,690 European-ancestry individuals from the UK Biobank (12). Genetic data for type 2 diabetes (T2DM) were obtained from the DIAMANTE Consortium GWAS meta-analysis of 80,154 cases and 853,816 controls, all of European ancestry (13). GWAS summary statistics for chronic kidney disease (CKD) were derived from the CKDGen consortium GWAS meta-analysis of 41,395 cases and 439,303 controls, all of European ancestry (14). Genetic association estimates for ischemic stroke were obtained from the GIGASTROKE Consortium GWAS meta-analysis of 62,100 cases and 1,234,808 controls, all of European ancestry (15). The cited original source GWAS publications provide population characteristics and trait definitions.

#### 2.1.2. Genetic instruments

To estimate the total effects of risk factors (SBP, DBP, BMI, WHR, LDL, TG, smoking, T2DM, and CKD) on ischemic stroke, genetic instruments were identified as single-nucleotide polymorphisms (SNPs) that were associated with the risk factor at genome-wide significance (*p* < 5 × 10^−8^) and were in pair-wise linkage disequilibrium (LD) *r*^2^ < 0.001. Instrument strength was estimated using the *F* statistic (16). The proportion of variance in the exposure explained by each genetic variant was calculated using the *R*^2^ value (17). To select genetic instruments for mediation analysis, the genetic variants associated with either primary exposure or the second exposure (mediator) at genome-wide significance were pooled and clumped to pair-wise LD *r*^2^ < 0.001. LD clumping was performed using R package TwoSampleMR (18). The effect alleles of genetic variants from different GWAS were aligned without exclusions for palindromic variants. Only variants that had genetic association estimates for the traits being investigated in any given analysis were included as instruments. Proxies were not used in the case of missing variants, in order to maintain consistency in the genetic variants selected as instruments across different analyses.

#### 2.1.3. Univariable MR analysis

MR analysis relies on three main assumptions: the genetic instruments are robustly associated with the exposure of interest; the genetic instruments are independent of potential confounders; and the genetic instruments directly affect the outcomes only via their association with the exposure.

To estimate the total effects of the risk factors on the odds of the outcome, a two-sample MR analysis using random-effect inverse-variance weighting (IVW) was performed as the main analysis. Estimates of the effects of each exposure on outcome are odds ratio (OR) per unit increase in genetically predicted exposure traits. A Bonferroni threshold of 0.005 that corrected for multiple testing was used to ascertain statistical significance in the main analysis, whereas *p*-value <0.05 but >0.005 was considered suggestive evidence. Weighted median, Mendelian Randomization-Pleiotropy Residual Sum and Outlier (MR-PRESSO), Causal Analysis Using Summary Effect estimates (CAUSE), MR Accounting for Pleiotropy and Sample Structure (MR-APSS), and MR Egger were used in the sensitivity analysis to assess the robustness of the results. Horizontal pleiotropy, which occurs when a genetic variant affects outcome independently of the pathway of the exposure, can lead to false-positive causal relationships in the MR analysis (19, 20). Horizontal pleiotropy can be categorized into uncorrelated pleiotropy (where a genetic variant affects exposure and outcome through separate mechanisms) and correlated pleiotropy (where a genetic variant affects both exposure and outcome through shared pathways) (21). The MR-PRESSO method detects potential horizontal pleiotropy using a regression framework in which the effects of variants on the outcome are regressed on the effects of the same variants on exposure. The method removes the outlier variants to correct for potential horizontal pleiotropy and subsequently performs the IVW method without such variants (20). The weighted median method provides a consistent estimate of the causal effect if at least 50% of the weight comes from valid genetic variants. The MR estimates from individual variants are ordered by their magnitude weighted for their precision, and the median of the variant-specific estimates was selected as the overall MR estimate (22). In the MR Egger method, the variant-outcome association estimates are regressed on the estimates of variant-exposure association, weighted for the precision of the variant-outcome estimates. The MR Egger method can detect horizontal pleiotropy and give a valid MR estimate, requiring the Instrument Strength Independent of Direct Effect (InSIDE) assumption. The InSIDE assumption is satisfied when the pleiotropic effects of the variants on the outcome are not correlated with their associations with the exposure (23). The CAUSE method accounts for both uncorrelated and correlated horizontal pleiotropy via a multivariable linear model adjusted by a joint distribution of genetic instruments, leveraging genome-wide summary statistics. To assess whether the data are consistent with a causal effect, the expected log pointwise density (ELPD) test is conducted to compare the level of fitness of the sharing model and the causal model (21). The MR-APSS method accounts for pleiotropy and sample structure using genome-wide summary statistics. MR-APSS performs causal inference based on a foreground-background model. The background model accounts for correlated pleiotropy, sample structure and polygenicity, and the foreground model performs causal inference while accounting for uncorrelated pleiotropy (24). Moreover, to detect potential population stratification, we estimated the effect of each phenotype under the study on self-reported tanning ability which was considered as a negative control outcome (25). GWAS summary statistics for tanning ability were derived from a GWAS on 453,065 individuals of European ancestry from UK Biobank, available through MR-Base (MR-Base id: ukb-b-533) (18).

The IVW, weighted median, and MR Egger analyses were performed using the “MendelianRandomization” package (version 0.6.0) in R (version 4.1.3) (26). MR-PRESSO was performed using the R package “MR-PRESSO” (20). CAUSE was performed using R package “cause” (21). MR-APSS was performed using R package “MRAPSS” (24).

### 2.2. Mediation analysis

Multivariable MR using GWAS summary data was performed to estimate the direct effect of exposure on the outcome, controlling for a potential mediator. Each of the nine investigated risk factors was considered as exposure, while each remaining risk factor was in turn considered as a potential mediator. The effect estimates for the instruments on the exposure, the mediator, and the outcome were harmonised by aligning effect alleles. The effects of variants on the outcome are regressed on the effects of the same variants on the exposure and on the mediator, weighted for the precision of the association between variant and outcome, with the intercept fixed to zero (27). The direct effect was subtracted from the total effect to estimate the indirect effect of the exposure on the outcome that acts via the mediator included in MVMR (28). The proportion of the total effect that is mediated was obtained by dividing the indirect effect by the total effect. All standard errors were estimated using the propagation of error method (28). In sensitivity analysis, to remove any bias that might be introduced due to binary exposures (29), genetically predicted glycated haemoglobin (HbA1c) and estimated glomerular filtration rate (eGFR) was used instead of genetic liability to T2DM and CKD, respectively.

MVMR analyses were performed using the “MendelianRandomization” package (version 0.6.0). All analyses were performed in R software version 4.1.3 (26).

### 2.3. Pathway enrichment analysis

Expression quantitative trait loci (eQTL) are genomic variants that are significantly associated with expression levels of one or more genes. From the selected genetic instruments of the considered risk factors, significant cis-eQTLs (*q*-value < 0.05) were identified by using Genotype-Tissue Expression (GTEx) Consortium database (version 8). GTEx consortium annotated genetic associations to gene expression for 54 non-diseased tissues across nearly 1,000 individuals (30). The list of cis-eQTL genes for each risk factor were analysed for enriched pathways using Reactome Knowledgebase (31). Reactome is a curated and peer-reviewed database of pathways and reactions in human biology. Over-representation analyses are conducted to determine whether specific Reactome pathways are enriched in the gene list, producing probability scores and the *p*-values corrected for false discovery rate (FDR) using the Benjamani-Hochberg method (31). The drugs involved in the significantly enriched pathways (FDR of 5%) were obtained and then classified using the Anatomical Therapeutic Chemical (ATC) system of the World Health Organization (WHO).

## 3. Results

### 3.1. Total effects

The study design and the number of selected genetic instruments for each risk factor were illustrated in Supplementary Figure S1. All the variants selected as genetic instruments had *F* statistics >10, corresponding to a <10% risk of bias and suggesting that bias due to weak instruments is unlikely to significantly affect the results. The total variance explained by the selected instruments is estimated as 4.52%, 4.72%, 4.93%, 3.24%, 6.17%, 5.40%, 1.20%, 0.29%, 1.55%, 4%, 2.6% for SBP, DBP, BMI, WHR, LDL, TG, smoking, CKD, T2DM, HbA1c, and eGFR, respectively. For all considered risk factors except CKD, the IVW MR showed that higher genetically predicted risk factor was significantly associated with an increased risk of ischemic stroke [SBP: OR, 1.307 (95% CI 1.260–1.356) per 10-mmHg; DBP: OR, 1.237 (95% CI 1.199–1.277) per 5-mmHg; BMI: OR, 1.192 (95% CI 1.131–1.256) per 1-standard deviation (SD); WHR: OR, 1.236 (95% CI 1.156–1.322) per 1-SD; LDL: OR, 1.089 (95% CI 1.04–1.141) per 1-SD; TG: OR, 1.089 (95% CI 1.035–1.145) per 1-SD; Smoking: OR, 1.342 (95% CI 1.206–1.493) per 1 unit lifetime smoking index score; T2DM: OR, 1.101 (95% CI 1.077–1.12) per logOR increase in T2DM liability]. Estimates obtained from the weighted median, MR-Egger, MR-PRESSO, CAUSE, and MR-APSS methods were consistent with the results from the main IVW analysis for all the significant risk factors except for TG (Supplementary Figure S2). The MR-Egger intercept did not provide evidence to suggest directional pleiotropy in any analysis except the analysis for SBP (*p* = 0.04) and TG (*p* = 0.01) (Supplementary Table S1). CAUSE indicated that the causal model was significantly better than a sharing model for all significant exposures except for LDL (ELPD *p*-value = 0.08) and TG (ELPD *p*-value = 0.39) (Supplementary Table S2). None of the phenotypes under the study reached the Bonferroni-corrected significant level in the MR analysis with self-reported tanning ability (Supplementary Table S3).

### 3.2. Mediation analysis

**Figure 1:**
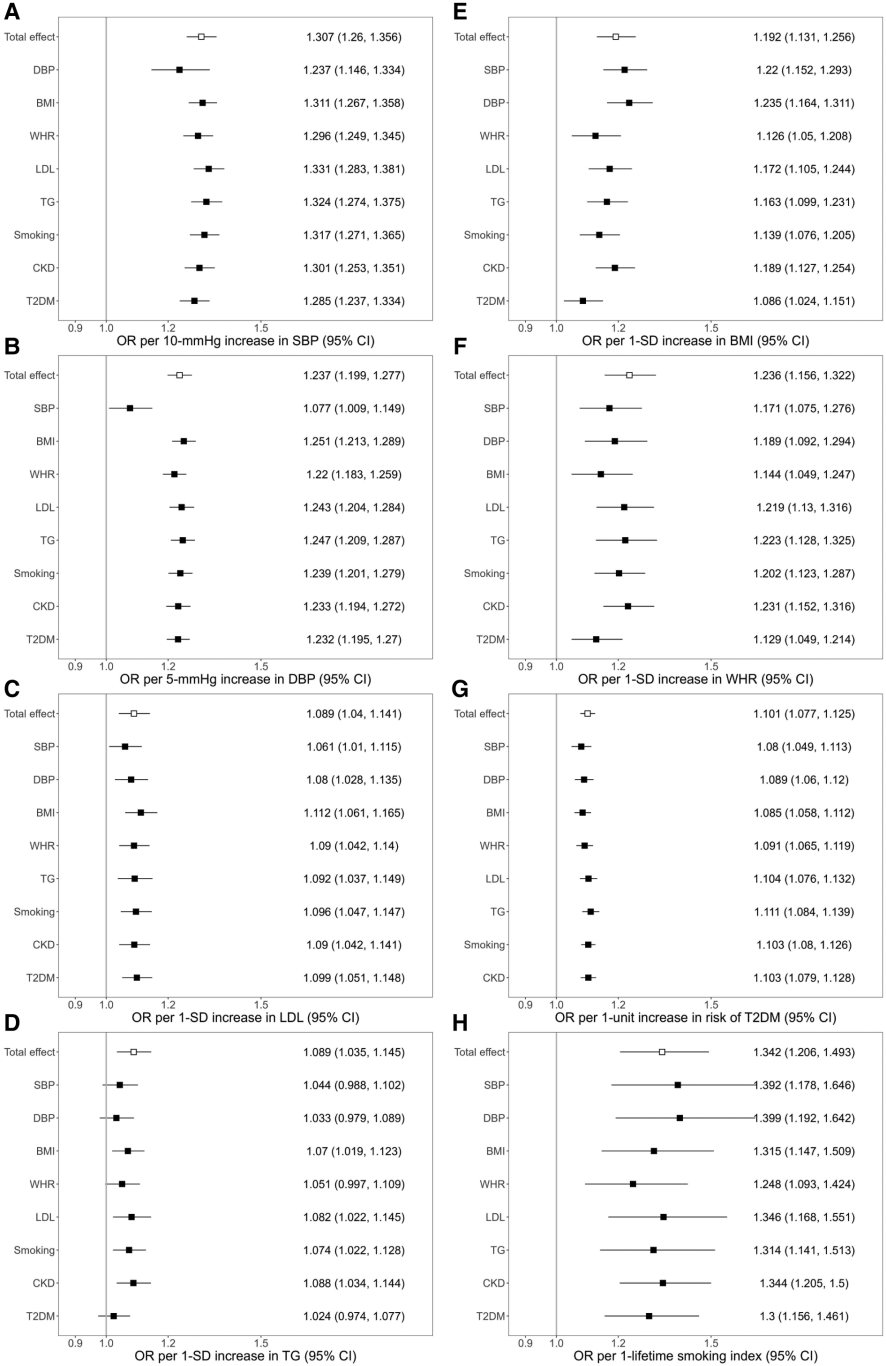
Associations of genetically predicted exposure [systolic blood pressure (**A**), diastolic blood pressure (**B**), serum low-density lipoprotein cholesterol (**C**), triglycerides (**D**), body mass index (**E**), waist-to-hip ratio (**F**), type 2 diabetes (**G**), smoking (**H**)] on ischemic stroke before and after adjusting for genetically predicted mediators. The *y*-axis details the genetically predicted mediator for which adjustments were made. SBP, systolic blood pressure; DBP, diastolic blood pressure; BMI, body mass index; WHR, waist-to-hip ratio; LDL, low-density lipoprotein cholesterol; TG, triglycerides; CKD, chronic kidney disease; T2DM, type 2 diabetes; OR, odds ratio; SD; standard deviation.

The association of genetically predicted SBP with ischemic stroke was attenuated after adjusting for genetically predicted DBP, while there was little change in the association after adjusting for other mediators. For a 10 mmHg increase in genetically proxied SBP, the OR of ischemic stroke decreased from OR, 1.307 (95% CI 1.260–1.356) to 1.236 (95% CI 1.146–1.334) after adjusting for genetically predicted DBP (Figure 1). In the association of genetically proxied DBP with the outcome, the OR was reduced from 1.237 (95% CI 1.199–1.277, per 5 mmHg) to 1.077 (95% CI 1.009–1.149, per 5 mmHg) after adjusting for genetically proxied SBP (Figure 1). There was attenuation in the association of genetically predicted BMI with the outcome after adjusting for genetically predicted WHR. The 19.2% (95% CI 13.1%–25.6%) increased risk of ischemic stroke per 1-SD increase in genetically predicted BMI decreased to 12.6% (95% CI 5%–20.8%) after adjusting for genetically predicted WHR. The association also attenuated after adjusting for LDL (17.2%, 95% CI 10.5%–24.4%), TG (16.3%, 95% CI 9.9%–23.1%), smoking (13.9%, 95% CI 7.6%–20.5%), CKD (18.9%, 95% CI 12.7%–25.4%), and T2DM (8.6%, 95% CI 2.4%–15.1%) (Figure 1). All considered mediators attenuated the association of genetically proxied WHR with the outcome (Figure 1).

The association of genetically predicted LDL and the outcome attenuated from OR 1.089 (95% CI 1.04–1.141) to 1.061 (95% CI 1.01–1.115) after adjusting for genetically predicted SBP, and to 1.08 (95% CI 1.028–1.135) after adjusting for genetically predicted DBP. Adjustments for each of all considered mediators led to the attenuation in the association of genetically predicted TG with ischemic stroke (Figure 1). In the association of genetic liability to T2DM with ischemic stroke, the OR decreased after adjusting for the BP traits, BMI, and WHR. In the association of genetically proxied smoking with the outcome, adjustments for BMI, WHR, TG, and T2DM led to the reduction of the OR of the outcome (Figure 1).

**Figure 2:**
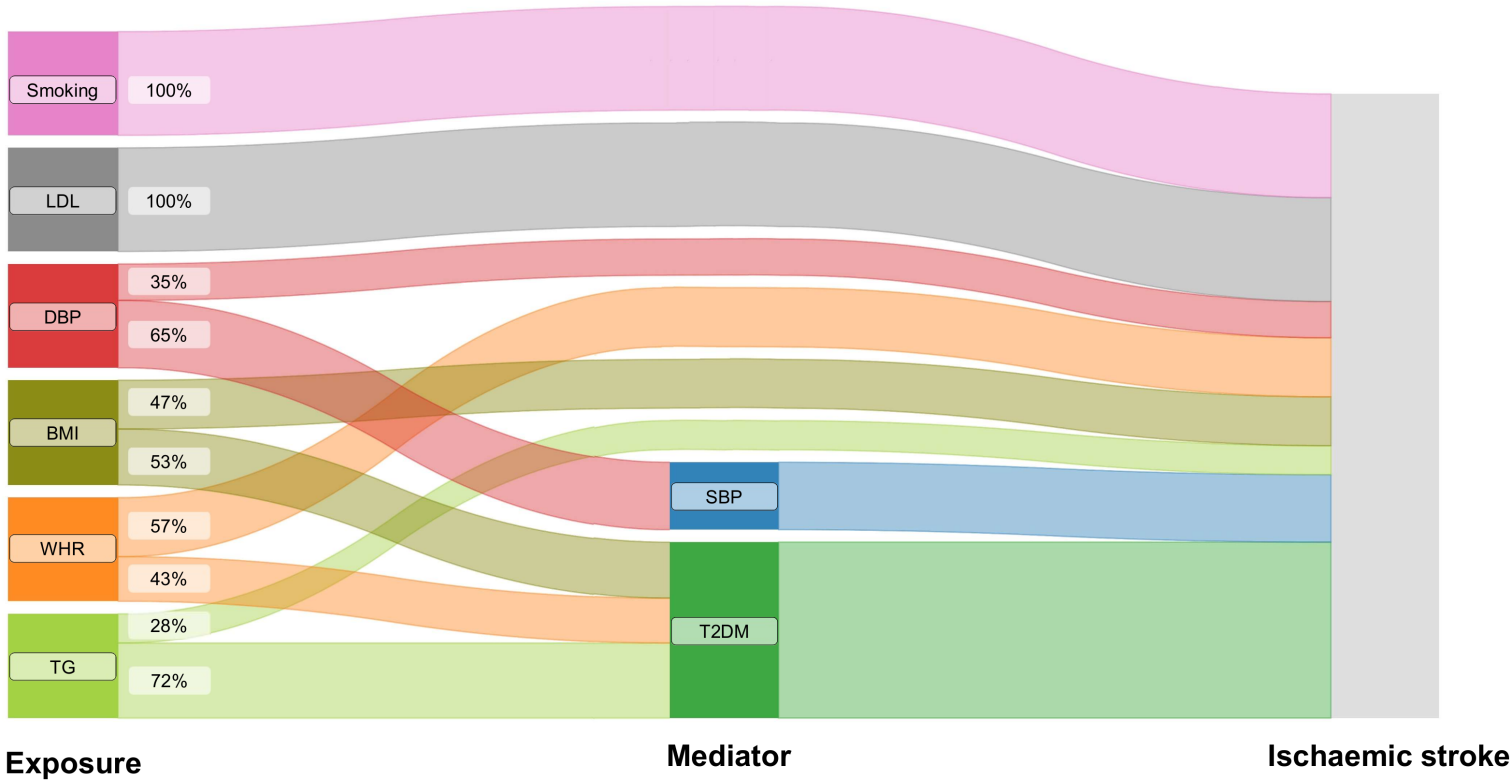
The effects of genetically predicted exposures on ischaemic stroke. The Sankey plot illustrates the direct and mediated effects of the risk factors on the risk of ischaemic stroke. SBP, systolic blood pressure; DBP, diastolic blood pressure; BMI, body mass index; WHR, waist-to-hip ratio; LDL, low-density lipoprotein cholesterol; TG, triglycerides; T2DM, type 2 diabetes.

MR mediation analyses found a significant mediation effect of genetically predicted SBP on the association of genetically predicted DBP with the risk of ischemic stroke (proportion mediated: 65.21%, 95% CI 34.36%–96.05%). The effects of genetically predicted BMI, genetically predicted WHR, and genetically predicted TG on the risk of outcome were significantly mediated by genetic liability to T2DM (for BMI, proportion mediated: 53.15%, 95% CI 17.21%–89.10%; for WHR, proportion mediated: 42.92%, 95% CI 4.17%–81.67%; for TG, proportion mediated: 72.05%, 95% CI 10.63%–133.46%) (Figure 2, Supplementary Table S4).

The results obtained from MR mediation analyses using genetically predicted eGFR and genetically predicted HbA1c instead of genetic liability to CKD and T2DM were generally consistent in direction with the results from the main analysis, although the proportion mediated by HbA1c was much smaller than the proportion mediated by T2DM (Supplementary Table S5).

### 3.3. Pathway enrichment analysis

**Figure 3:**
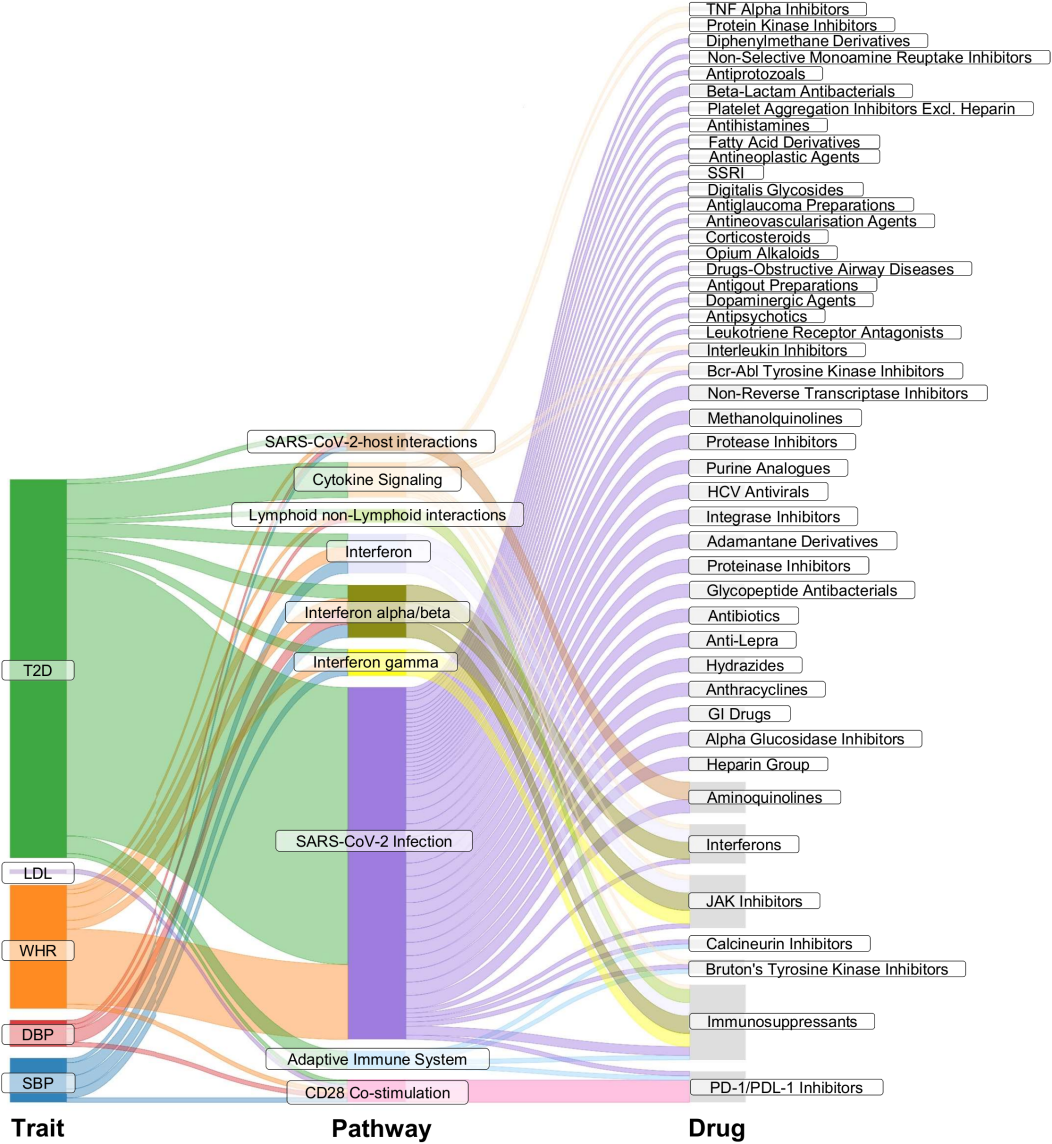
Pathway enrichment analysis. The Sankey plot illustrates enriched pathways (middle column) in the list of genes that had expression levels associated with the genetic instruments for the considered exposure traits (left column). The right column details drugs involved in the enriched pathways. SBP, systolic blood pressure; DBP, diastolic blood pressure; WHR, waist-to-hip ratio; LDL, low-density lipoprotein cholesterol; TG, triglycerides; T2DM, type 2 diabetes. SARS-CoV-2, severe acute respiratory syndrome coronavirus 2; TNF, tumor necrosis factor; SSRI, selective serotonin reuptake inhibitor; HCV, hepatitis C virus; GI drugs, gastrointestinal drugs; JAK Inhibitors, Janus kinase inhibitors; PD-1/PDL-1, programmed cell death protein 1 and programmed death ligand 1.

Reactome pathway enrichment analysis revealed significant enrichment (*p* < 0.05, FDR of 5%) of SBP eQTL-related genes (i.e., genes that have the expression levels associated with the instruments for SBP, FDR < 0.05) in pathways involved in (1) class I major histocompatibility complex (MHC) mediated antigen processing and presentation; (2) costimulation by the CD28 family; (3) interferon (alpha/beta, gamma) signalling; (4) SARS-COV-2-host interactions; and (5) T cell receptor (TCR) signalling. Genes related to DBP eQTLs, WHR eQTLs, and T2DM eQTLs resulted in the same enriched pathways as those for SBP, with extra pathways involving in (6) immunoregulatory interactions between a Lymphoid and a non-Lymphoid cell; (7) SAR-CoV-2 infections (WHR, T2DM); (8) adaptive immune system (T2DM); (9) cytokine signalling in the immune system (T2DM); (10) MHC class II antigen presentation (T2DM); (11) oncogene-induced senescence (T2DM). The enriched pathways in LDL eQTL-related genes involved in costimulation by the CD28 family and TCR signaling; NR1H2 and NR1H3-mediated signalling were enriched by TG eQTL-related genes. The enriched pathway in the CKD eQTL-related genes was elastic fibre formation, and for smoking eQTL-related genes the enriched pathway was acetylcholine binding and downstream events (Figure 3, Supplementary Table S6). Figure 3 illustrates the enriched pathways that had drugs participating in, Supplementary Table S6 details all pathways that were significantly enriched in the gene lists of the considered risk factors (*p* < 0.05, FDR of 5%).

JAK inhibitors (ruxolitinib, baricitinib, tofacitinib), interferons (interferon alpha, interferon beta), PD-1 and PD-L1 inhibitors (cemiplimab, nivolumab), selective immunosuppressants (emapalumab, sirolimus, mycophenolic acid), and aminoquinolines (hydroxychloroquine, chloroquine) were found to be involved in the enriched pathways by the genes related to instruments for SBP, for DBP, for WHR, and for T2DM. The PD-1/PD-L1 inhibitors were also involved in the enriched pathways by the eQTL-related genes for LDL (Figure 3).

## 4. Discussion

Leveraging large-scale genetic association data within the MR paradigm, this study investigates the direct and indirect effects of nine risk factors on ischemic stroke. The results show that genetic predisposition to higher SBP, DBP, BMI, WHR, LDL, TG, T2DM, and smoking index is associated with a higher risk of ischemic stroke in concordance with previous MR (32–36) and epidemiology studies (37–42). The effects of BMI and WHR on ischemic stroke are found to be significantly mediated by T2DM. Both genetically predicted BMI and genetically predicted WHR have direct associations with ischemic stroke after inclusion in the same model, albeit with attenuation in the associations. These findings are consistent with the previous work (43). The association of genetically predicted DBP with ischemic stroke is attenuated significantly after adjusting for genetically proxied SBP, which is also supported by a previous study (32). This study also supports that T2DM is a significant mediator in the association between TG and the outcome. To mitigate the bias that might be introduced due to the binary exposure (29), the sensitivity analysis using genetically predicted HbA1c instead of genetic liability to T2DM was conducted. The results were generally consistent with the main analysis albeit smaller proportion mediated, which may be because T2DM has manifestations other than just impaired glycaemic control. Findings from pathway analysis show that drugs from five drug classes (JAKis, interferons, PD-1/PD-L1 inhibitors, selective immunosuppressants, and aminoquinolines) are involved in enriched pathways in the genes related to instruments for SBP, DBP, WHR, and T2DM. PD-1/PD-L1 inhibitors are also involved in the enriched pathways by the eQTL-related genes for LDL. Some of these drugs have been previously found to be associated with the risk of ischemic stroke, highlighting the applicability of our method in flagging potential drugs with adverse events for a selected condition. JAKis, which are used to treat chronic inflammatory disorders, have been found to increase the risk of MACE, venous thromboembolism, malignancy, and serious infections (7). EMA advised that all commercially available JAK inhibitors (Xeljanz, Cibinqo, Olumaint, Rinvoq and Jyseleca) used for the treatment of chronic inflammatory disorders should only be prescribed when there's no suitable alternative (5). ICIs, which are monoclonal antibodies targeting cytotoxic CTLA-4, PD-1/PD-L1, and LAG-3, were associated with higher risk for cardiovascular events, potentially mediated by accelerated atherosclerotic plaque progression, and the promotion of plaque stability (1).

Our study has some limitations. The validity of MR relies on three major assumptions: the genetic instruments were strongly associated with the exposure of interest; the genetic instruments were independent of potential confounders; and the genetic instruments directly affected the outcomes only via their association with the exposure. In this study, the genetic instruments were strongly associated with the exposure traits at genome-wide significance (*p* < 5 × 10^−8^), and all of them had *F* statistic >10, suggesting that the first assumption is likely satisfied. Although the second and the third assumptions cannot be fully verified, the concordance in direction and magnitude of MR estimates for the total effect of the exposures on the outcome across the sensitivity analysis indicates the robustness of the findings to potential pleiotropic effects of the variants (44). The MR estimates for the exposures under study are consistent across the sensitivity analysis (CAUSE, MR-APSS, MR-PRESSO, MR-Egger and weighted median methods) except for TG, where potential pleiotropic bias was indicated in the TG-ischemic stroke association as the MR estimate obtained from the weighted median method was inconsistent with the IVW MR estimate. Negative control outcome was also used to detect potential population stratification which can lead to the violation of the MR assumptions (25). None of the considering phenotypes reached the Bonferroni-corrected significant level in the MR analysis with self-reported tanning ability, suggesting that bias due to population stratification is unlikely to significantly affect the results. The analyses in this study use GWAS summary statistics drawn from the European population and thus may not apply to other populations. Finally, Reactome overrepresentation analyses are conducted by overlaying the experimental dataset on annotations (31). Certain bias into the statistical analysis may be caused because certain areas of biology are annotated with more details and more accurate terms for well-known processes than others (45).

## 5. Conclusions

By using the MR framework this study highlights the causal effects of metabolic risk factors on the risk of ischaemic stroke and the role of T2DM in mediating the effects of BMI, WHR, and triglycerides. Recently the FDA and EMEA have flagged serious cardiovascular adverse events for JAK inhibitors and PD-1/PD-L1 inhibitors, both of which have appeared in our pathway enrichment analysis using eQTLs. This supports a potential utility of this approach in early identification of harm from drugs, thereby informing clinical trial design and pharmacovigilance studies to anticipate any unexpected adverse events.

## Data Availability

The original contributions presented in the study are included in the article/**Supplementary Material**, further inquiries can be directed to the corresponding author.
